# Community detection framework based on 3D shape descriptors for tree species classification in point cloud data

**DOI:** 10.1038/s41598-026-42392-4

**Published:** 2026-03-04

**Authors:** Štefan Kohek, Borut Žalik, Domen Mongus, Damjan Strnad

**Affiliations:** Faculty of Electrical Engineering and Computer Science, University of Maribor, Koroška cesta 46, SI-2000 Maribor, Slovenia

**Keywords:** Tree species classification, Community detection, Algorithm, Remote sensing, Point clouds, Feature vectors, Forestry, Computational science, Computer science

## Abstract

Accurate tree species classification from remote sensing data, such as LiDAR point clouds, is important for various applications, including vegetation monitoring and forest growth prediction. Although numerous machine learning algorithms are used widely for these tasks, several challenges remain. These include the need for extensive training datasets, over-fitting to specific geographic areas and environmental conditions during tree growth, the requirement for post-processing adjustments, and unreliable performance with rare tree species and shapes. This paper presents a robust framework for classifying tree species directly from diverse point cloud datasets, eliminating the need for training machine learning models or manually preparing the training datasets. The proposed approach performs community detection on a graph using features extracted from point clouds of individual trees. Multiple shape descriptors are proposed as feature vectors that consider 3D tree crown structure and are rotationally invariant. Community detection groups the trees into distinct communities based on these feature vectors. Tree species classification is performed by classifying these communities, reducing the manual effort significantly, as only a few trees from each community need inspection. The proposed framework was validated using both real-world terrestrial LiDAR and synthetic point clouds. The results demonstrate its competitiveness with established methods, while surpassing the performance of traditional clustering techniques applied to the same feature vectors. Additionally, the results confirm the effectiveness of the proposed feature vectors for achieving competitive tree species classification accuracy.

## Introduction

Tree species classification is an important step in building accurate and detailed tree inventories (e.g. forest and urban vegetation inventory), which are necessary for efficient planning of tree management. Proper tree management is essential for enhancing the positive impacts of trees on the environment (e.g., improving microclimate conditions^1^, mitigating landslides^2^, or reducing erosion^3^) and mitigating the negative ones (e.g., damaging infrastructure^4^). In that context, tree species information is important for inferring tree growth rate and shape parameters, enabling advanced analyses, such as biomass estimation^5,6^, and growth prediction^6–8^.

Tree forest inventory data can be gathered manually in the field, however, large-scale areas require automatic mapping from remote sensing data^9^. Commonly used remote sensing data sources for vegetation and forests include satellite data^10^, which enable large-scale analyses. However, the main drawback of freely accessible satellite data is their insufficient resolution for extracting details at the level of individual trees. An alternative remote sensing data source for managing forest inventory data is airborne topographic LiDAR, where 3D point clouds provide information about tree structure at a resolution ranging from a few points to hundreds of points per m$^2$^11^. The emerging trend of using unmanned aerial vehicles (UAVs) provides a more affordable^12^ source of point cloud data for vegetation monitoring^13^.

Point clouds allow automatic detection of trees and their parameters^14–16^. The most commonly derived parameters of individual trees are their 3D location, height, and crown width, while more precise analyses can benefit from knowing the detailed 3D tree crown shapes^8,17^. One of the tree parameters that are challenging to extract is the tree species^18,19^. Determining tree species requires assessing detailed tree shape, not only its height, from noisy and often sparse point clouds. Additionally, the trees of the same species can exhibit high variability of shape, especially in the presence of outliers, such as damaged or dead trees.

Several methods have been proposed for classifying tree species based on point cloud data^18,19^. The most common approach to species classification involves three steps: delineation of individual trees from point clouds^14,16^, extraction of individual trees’ features (such as the ratio between tree height and crown width), and classification of the derived features to species based on supervised machine learning algorithms. This workflow has several challenges, e.g., the need for extensive training datasets, over-fitting to specific geographic areas and environmental conditions during tree growth, the requirement for post-processing adjustments, and unreliable performance with rare tree species and shapes.

This paper proposes a complete framework for tree classification. While the methods based on supervised machine learning typically produce area-specific classification models, and require larger training datasets, the proposed framework can be applied directly on diverse point clouds without any computationally demanding preprocessing. The proposed framework first extracts the features for each tree, based on its shape, such as a measure of rotational symmetry through the vertical axis, represented by a fitted curve. These features are shape descriptors, and are invariant to point cloud rotations and robust to variations in density. The next step generates a graph, where trees represent the nodes, while the weights of the edges connecting the nodes correspond to the distances between the corresponding trees’ features. As the features in each pair of trees are independent and have diverse scales and ranges, they are combined into a single scalar distance using ranking-based differences. Subsequently, community detection is performed on this graph of features to group the trees by species. These steps are automated, without any preparation of a learning dataset. The final tree species classification is achieved by manual labelling of these communities into classes, where only one or a few trees from each community need manual inspection. This approach allows easy identification of smaller communities (i.e., outliers), representing, for instance, dead trees. Furthermore, diverse shapes resulting from various environmental conditions and tree properties can be inspected manually on just a few instances from each community. This eliminates the need for preparing larger learning datasets, which is the typical requirement for supervised machine learning methods. The proposed framework offers good interpretability of results compared to many machine learning methods that often behave as black boxes. Therefore, the main contributions of this paper are:

- A new framework for tree species classification, based on new 3D shape descriptors for tree features and community detection.
- New rotationally invariant 3D shape descriptors that capture detailed tree shapes.
- A method for representing tree shape descriptors using fitted curves.
- The transformation of tree features into a graph for community detection using ranking-based distances.

The proposed framework was validated using two publicly available datasets with varying point cloud densities. Tree species classification performance was evaluated using metrics derived from community detection results and compared with PointConv^20^. Additionally, the proposed tree features were assessed for classification accuracy using Support Vector Machines (SVM).

The following section offers a more detailed overview of related work, while section 3 describes the proposed framework and the datasets used. In section 4, the proposed framework is validated, including a comparison with related methods and an analysis of the impact of various parameters on the clustering performance.

## Related work

Species classification based on feature extraction supports the use of features from multiple data sources. Previous work has classified tree species using LiDAR data alone or in combination with hyperspectral imagery^21–24^, multispectral data^25,26^, or aerial photography^27^. For example, classification accuracy has been increased by up to 10% when augmenting hyperspectral imagery with LiDAR data^21^, demonstrating that high-density point clouds are crucial for achieving good performance^21^. However, combining multiple data sources is often difficult. Due to the inverse relationship between spatial and spectral resolution^28^, satellite-based multispectral and hyperspectral data often have a low resolution (typically more than 1 meter per pixel). This makes satellite data unsuitable for identifying individual trees and more useful for classifying large, homogeneous forests where individual tree characteristics are less important. Although UAVs can be equipped with multispectral/hyperspectral sensors to capture imagery at a much higher resolution of just a few centimetres per pixel^29^, they can typically cover only limited areas. Other data sources, such as synthetic-aperture radar (SAR)^30^, typically have a resolution that is too low for effective tree species classification.

Tree species classification from LiDAR data can utilise geometric, radiometric (intensity and reflectivity) and waveform information^19^. Combining these types of data can lead to higher accuracy. However, previous studies have identified a high influence of the sensor properties on accuracy^31^. Therefore, the same method may have problems when using different sensors or performing scanning in different conditions (e.g. atmospheric attenuation and flying altitude), requiring radiometric calibration^32^. Moreover, radiometric data is not always available, making the 3D structure the most reliable source of information.

The most frequently used features, derived from LiDAR point cloud data for tree species classification, include normalised tree height, crown penetration depth, height percentiles, density through vertical slices of the tree crown^32^, and other statistical measures (e.g., skewness and kurtosis of the laser height distributions)^21,32^. Lee et al.^33^ used additional feature post-processing algorithms, such as principal component analysis (PCA), to improve accuracy. In terms of geometry processing, there remain opportunities to extract even more data from point clouds. Therefore, PCA can be applied directly to terrestrial LiDAR point clouds to delineate the branching structure of a conifer tree and derive more complex geometric features^17^, providing a detailed tree shape description for species classification.

Environmental growth conditions have a major impact on the shape of a tree’s crown^34^, but they affect different tree species in unique ways. The 3D shape of trees can be described parametrically with compact shape descriptors, such as alpha shapes^8^ and cones^17^. Although the 3D shape has a major role in tree species identification, detailed 3D shape descriptors have been used rarely in previous work. Shape descriptors should reflect typical features of tree species and variations in shape throughout the tree crown. Symmetry-based shape descriptors^35^ have proven to be effective in more general classification and shape retrieval^36^. Even in recent years, new methods are still being developed for more efficient symmetry detection^37^. Therefore, symmetry-aware shape descriptors are worth investigating for tree species classification.

In tree species classification, the derived features are used most commonly as input for numerous classification algorithms. Some of the popular algorithms include logistic regression^38^, linear discriminant analysis^39^, random forests (RF)^21,40^, support vector machines (SVM)^21,39,40^, artificial neural networks^40^, and ensemble methods^41^. Because no single classification algorithm is universally optimal, some studies on species classification have verified and compared multiple algorithms. For instance, Dalponte et al.^21^ conducted species classification for a forest with 7 species on hyperspectral imagery and LiDAR data, finding that SVM achieved an 83% overall accuracy, thereby outperforming random forests (80% overall accuracy). Raczko and Zagajewski^40^ demonstrated improved classification accuracy using artificial neural networks with overall classification accuracy of 77% while SVM and RF achieved 68% and 62%, respectively. While machine learning algorithms can achieve high accuracy, they require careful tuning of the model architecture and training hyperparameters.

Features are typically engineered manually, and then used as input for classification methods like random forests. In contrast, deep neural networks can be applied directly to point cloud data to learn features implicitly. Seidel et al.^42^ evaluated two distinct neural network architectures: PointNet and convolutional neural networks (CNNs). While PointNet processes point clouds directly, CNNs require structured grid data. Consequently, Seidel et al.^42^ projected point clouds onto multiple 2D image representations and improved the accuracy using CNNs from about 48% overall acuracy to 80.2% and even 86.01% with data augmentation. Subsequently, Liu et al.^43^ compared various neural network architectures for tree species classification (PointNet, PointNet++, PointMLP, PointConv, graph-based DGCNN, attention-based PCT), demonstrating that PointConv achieved the highest overall accuracy of 99.5%, while PointNet was the worst with 72.9%. However, careful interpretation of these results is necessary because they were not obtained on the same datasets, and it is therefore better to focus on the differences in accuracies within each study.

Most species classification methods based on supervised machine learning have been validated on specific point clouds from limited areas, particular time periods (e.g., summer), and homogeneous forests with uniform environmental conditions. However, trees in a natural environment have more diverse shapes, influenced not only by genotype, but also by age, season, global environmental conditions and phenotype^44^. An obvious difference in tree shape exists between trees in the forest interior and those at its edge. Environmental conditions and competition affect tree shape significantly^34^, which can, consequently, decrease the accuracy of species classification based on a limited training dataset. Moreover, it remains unclear whether outliers (e.g., dead or pruned trees) should be classified as a separate class, as these outliers can impact the accuracy of supervised machine learning models negatively.

A fundamental challenge of supervised machine learning-based methods is their strong dependency on the training dataset. These classification algorithms require substantial amounts of high-quality training data, which requires manual preparation in advance, thus limiting the usability of these methods. Furthermore, inadequate training datasets containing noise and imbalanced classes can lead to the accumulation of classification mistakes^45^. Michałowska and Rapiński^19^ demonstrated that even an increase in the number of discriminated species can result in a decline in classification accuracy. Therefore, even a change in the number of tree species might require adaptations of the classification models.

Despite their advantages, classification methods based on supervised machine learning necessitate manual preparation of training datasets and inspection of the results. Alternatively, tree species classification can be achieved without the need for training data preparation through unsupervised clustering analysis^46^. This approach groups species into clusters based on derived features. Subsequently, these clusters can be classified efficiently into species by identifying a single tree manually from each cluster and labelling the remaining trees within that group automatically.

Previous work on clustering analysis for species classification^46^ is limited, due to several challenges that need to be addressed. The most common clustering algorithms require a predefined number of clusters (e.g., k-means) or predefined threshold values (e.g., hierarchical clustering), which demands prior knowledge regarding the expected species. Furthermore, the resulting clusters often need manual verification to optimise the clustering hyperparameters. Lastly, the extracted features can have different scales, potentially impacting clustering performance^47^.

A more recent and promising alternative to traditional clustering is community detection^48^. This method operates on graphs, grouping entities into communities based on the distance between nodes. It identifies groups of nodes with dense internal connectivity effectively. Community detection has been applied to large-scale networks successfully^49,50^. However, applying traditional feature-based clustering approaches to community detection requires an appropriate transformation of the problem into a graph structure.

## Methodology

**Figure 1:**
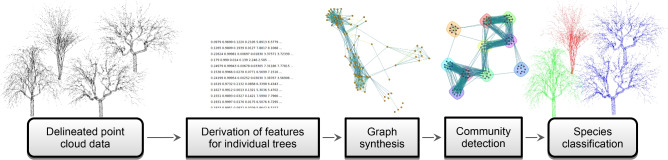
Overview of the proposed tree species classification.

The proposed framework for tree species classification consists of the steps outlined in Fig. 1 and detailed in the subsequent subsections.

The input is a point cloud delineated into individual tree point sets^14,16^. In the next step, feature vectors are generated from the point sets of individual trees. The proposed features are designed to be rotationally invariant and to account for local shape variance. The distances between trees are calculated based on the derived features. Given that the feature vectors are represented in diverse scales, this paper proposes ranking-based distances. These distances are used for graph construction, where the trees with the features represent the nodes, and the distances are transformed into edge weights. The graph is subsequently used for community detection to identify community membership. Finally, the proposed framework utilises the memberships of trees for the final species classification step, which can be performed automatically or semi-automatically.

### Feature extraction

The feature extraction step processes the delineated point cloud $\textrm{L}_t$ for each tree *t* individually to derive a feature vector $\mathrm {F^{(t)}} = \left\{ \vec {f}^{(t)}_1, f^{(t)}_2, \vec {f}^{(t)}_3, \vec {f}^{(t)}_4, \vec {f}^{(t)}_5, f^{(t)}_6 \right\}$ for each tree *t*, where *f* and $\vec {f}$ can be either a scalar or a vector, respectively. The derived features describe the tree shape in a concise way for robust species discrimination. While previous methods have already employed as features some statistical characteristics of the point clouds, such as the variance of all height points^21^, the proposed method introduces more detailed shape descriptors, which are, at the same time, invariant to tree rotation and robust to variance in the point cloud density. Additionally, a symmetry measure is proposed, because it is affected by growth conditions, to which different species respond in unique ways^34^. The proposed features are described in more detail as follows.

The first feature is derived by using the principal component analysis (PCA) directly on the point clouds of individual trees. Previous work has shown that PCA can provide good separability between tree species^17^. However, validation in the previous studies was often conducted on homogeneous forests with uniform growth conditions (e.g., even shading from neighbouring trees which promotes symmetric tree crown). In contrast, the proposed approach aims to support heterogeneous environmental growth conditions, which cause asymmetric tree shapes by incorporating rotational invariance. To make the feature robust to these variations, we first perform the PCA directly on the 3D coordinates of the point cloud $\textrm{L}_t$. The PCA calculates three eigenvectors, which define 3D principal component directions of the tree’s shape. In the next step, we calculate the standard deviations $d_i$ of point coordinates along each of the principal directions $i \in {1,2,3}$. Finally, we use the pairwise differences between these standard deviations as the components of the first feature vector $\vec {f}_1^{(t)} = \left[ d_1 - d_2, d_1 - d_3, d_2 - d_3\right]$.

While PCA-derived features are valuable shape descriptors, they do not capture the exact shape of the tree and local variations within it. Therefore, more detailed shape descriptors that remain rotationally invariant are proposed as remaining feature vectors. For this purpose, a voxel grid $\text {V}$ is constructed with the isotropic voxel size $\delta$, which is a user-defined parameter. Based on a visual analysis of the point cloud density, a default voxel size of 0.5 m was chosen for the experiments. This size was empirically found to be the optimal trade-off that preserved tree crown connectivity without gaps, but still allow the voxel grid to accurately represent the crown shape.

Each voxel $v_{i,j,k} \in \text {V}$ is an integer value representing the number of points from $\text {L}_t$ that are residing within that voxel. The central position $\vec {c}$ of $\text {V}$ is calculated as the centre of mass of all points $\vec {c} = \frac{1}{|\textrm{L}_t|} \sum _{l \in \textrm{L}_t} l$. Here, $|\textrm{L}_t|$ represents the number of points in the point cloud $\textrm{L}_t$.

**Figure 2:**
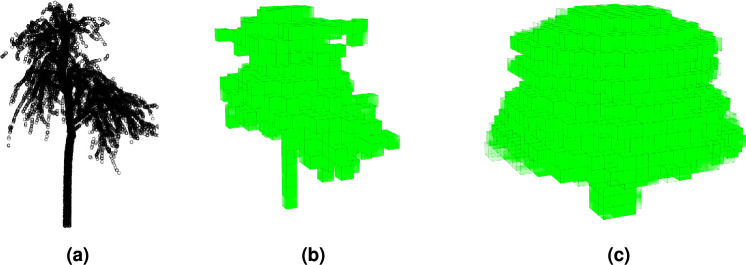
(**a**) Example of an individual tree point cloud from a publicly available dataset^51^, (**b**) corresponding voxel grid V, and (**c**) corresponding rotationally symmetric grid S.

In the next step, a symmetric grid $\text {S}$ is generated by symmetrising $\text {V}$. This is achieved similarly to creating a surface of revolution, i.e., rotating the point cloud $\text {L}_t$ around the vertical (*z*) axis passing through the centre, $\vec {c}$, and accumulating the points into the visited voxels $s_{i,j,k} \in \text {S}$. Specifically, point $\vec {l} \in \text {L}_t$ is counted in voxel *s* if $||\vec {l}_{xy}-\vec {c}_{xy}|- |\vec {s}_{xy}-\vec {c}_{xy}||< \delta$ and $|\vec {l}_{z}-\vec {s}_{z}|< 0.5 \delta$, where $\vec {s}_{xy}$ and $s_{z}$ represent the horizontal and vertical components of the centre of voxel *s*, respectively. Rotationally symmetric voxelised tree representation is produced in this way. An example of such a grid is shown in Fig. 2.

The symmetric grid S is used to derive the remaining features $f_2^{(t)}$ - $f_6^{(t)}$. Feature $f_2^{(t)}$ is calculated as the Jaccard index between the binarised grids $S^{(b)}$ and $V^{(b)}$:

1 $$\begin{aligned} f^{(t)}_2 = \frac{|V^{(b)} \cap S^{(b)}|}{|V^{(b)} \cup S^{(b)}|}, \end{aligned}$$

where, $\text {V}^{(b)}$ and $\text {S}^{(b)}$ are binarised representations of the voxel grids $\text {V}^{(b)}$ and $\text {S}^{(b)}$, respectively. Each voxel $v^{(b)}_{i,j,k} \in \text {V}^{(b)}$ and $s^{(b)}_{i,j,k} \in \text {S}^{(b)}$ is assigned a binary value based on the presence of points in the original grids:

2 $$\begin{aligned} & v^{(b)}_{i,j,k} = \min \left( 1, v_{i,j,k} \right) , \end{aligned}$$

3 $$\begin{aligned} & s^{(b)}_{i,j,k} = \min \left( 1, s_{i,j,k} \right) . \end{aligned}$$

Therefore, $f^{(t)}_2$ is a measure of the rotational symmetry of the tree shape. Because $\text {V}^{(b)} \subseteq S^{(b)}$, $f^{(t)}_2 = \frac{|V^{(b)}|}{|S^{(b)}|}$.

Feature vector $\vec {f'}^{(t)}_3$ contains the rotational symmetry values of the tree’s horizontal slices by calculating the Jaccard indices (equation (1)) between the corresponding horizontal slices of S and V. The dimensionality of $\vec {f'}^{(t)}_3$ corresponds to the tree height divided by the voxel size. The next two features are also feature vectors: $\vec {f'}^{(t)}_4$ contains the number of points per horizontal slice of S, and $\vec {f'}^{(t)}_5$ is the maximum distance of points from the central vertical axis per horizontal slice of S.

To maintain the comparability of features across trees, the dimensionality of feature vectors $\vec {f'}^{(t)}_3$ through $\vec {f'}^{(t)}_5$ must be consistent. Therefore, cubic smoothing splines^52^ are fitted to each of $\vec {f'}^{(t)}_3$ through $\vec {f'}^{(t)}_5$, resulting in new final features $\vec {f}_3^{(t)}$ through $\vec {f}_5^{(t)}$. Each new feature contains 6 fitted coefficients of cubic spline basis functions, which makes the feature robust to noise and potential outliers in the point cloud. The number of coefficients was determined through preliminary experiments to provide a good balance between fitting the data accurately and maintaining smoothness.

**Figure 3:**
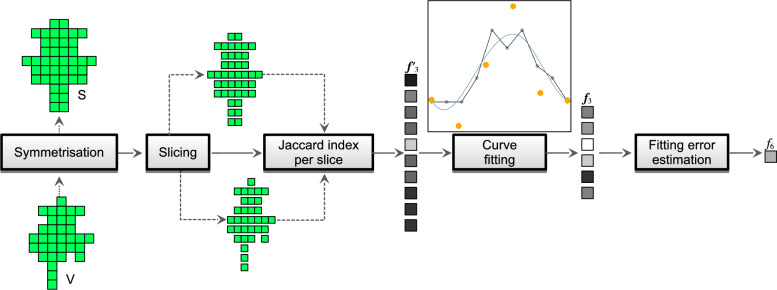
Construction of features $\vec {f}_3$ and $f_6$, where solid arrows represent the execution workflow, while dashed arrows represent the data flow..

Since curve fitting introduces errors, the absolute sum of fitting differences for each $\vec {f}_3^{(t)}$ through $\vec {f}_5^{(t)}$ could be a useful feature. Based on our experiments with the proposed approach, fitting errors of $\vec {f}_3^{(t)}$ were sufficient, therefore, we included them in $\text {F}^{(t)}$ as $f_6^{(t)}$. Fig. 3 outlines a workflow example for deriving features $\vec {f}_3$ and $f_6$ through curve fitting.

### Detection of species communities

This paper proposes community detection^53^ for grouping trees with similar characteristics into species. Because community detection works on graphs, trees with features need to be transformed into nodes and edges. In the proposed approach, each tree with its features represents a node in the graph. The distances between the features of different trees define which nodes are connected. The calculation of distance is rank-based, as follows. First, for every pair of trees $u,v \in T$, the distance between corresponding features $f^{(u)}$ and $f^{(v)}$ is computed as:

4 $$\begin{aligned} d(f^{(u)},f^{(v)}) = {\left\{ \begin{array}{ll} |f^{(u)} - f^{(v)}|& \text {if } f^{(u)} \text { and } {f}^{(v)} \text { are scalars}, \\ 1 - \frac{\vec {f}^{(u)} \cdot \vec {f}^{(v)}}{\Vert \vec {f}^{(u)} \Vert \cdot \Vert \ \vec {f}^{(v)} \Vert }, & \text {otherwise}. \end{array}\right. } \end{aligned}$$

Individual features are on different scales; therefore, the calculated distances are ranked for each individual feature, which is a common practice in other applications^54^:

5 $$\begin{aligned} R_{f}(u, v)= \text {rank}_{D_f}(d(f^{(u)},f^{(v)}) | u,v \in \textrm{T} ), \end{aligned}$$

where

6 $$\begin{aligned} D_f = \{ d(f^{(x)},f^{(y)}) | x,y \in \textrm{T} \}. \end{aligned}$$

Function $\text {rank}_{D_f}$ uses standard competition ranking and returns the rank for feature *f* within the set $D_f$ of all possible pairwise distances for feature *f* across all the trees in T.

Finally, each weight between trees/nodes *u* and *v*, $w_{u, v}$, is calculated as the product of the inverse rank order distances per each feature $f \in \textrm{F}$:

7 $$\begin{aligned} w_{u, v}=w_{v, u}=\prod _{f \in \textrm{F}}\left( 1- \frac{R_{f}(u,v)}{\max _{x, y \in \textrm{T}}\left( R_{f}(x,y)\right) }\right) ^\alpha , \end{aligned}$$

where $u \in \textrm{T}$ and $v \in \textrm{T}$, and $\textrm{F}$ is the set of all features. The value $\alpha$ modulates the differences between the larger and smaller weights, i.e., influences the number of desired communities. In our experiments, $\alpha$ was set to 2.

In the next step, $w_{u, v}$ is assigned to edges to construct the undirected graph $G=(V, E)$. In the proposed method, the $\beta$ share of edges with the smallest weights are discarded, to emphasise the characteristics of dense subgraphs with more edges within communities than between them. Higher pruning threshold facilitates detection of outliers as they become isolated, unconnected to the remaining trees. In our experiments, $\beta$ was set to 0.1.

Based on the generated undirected graph with established network topology and edge weights, community detection is performed using the parameter–less fast greedy modularity optimisation algorithm^55^. The result of community detection for each tree *t* is the index $M_t$ of the community to which the tree belongs. Efficient community detection algorithm, which considers weights of edges, should have high success rate with $\beta = 0$. However, some community detection algorithms work without edge weights by using only the graph topology. In such cases, higher $\beta$ around 0.9 would support community detection while $\alpha$ would be unused.

### Tree species classification

The main purpose of the proposed framework is automatic or semi-automatic tree species classification. Therefore, the community membership of each tree $M_t$ is used for subsequent tree species classification by labelling at least one tree within each community manually. In the proposed framework, the species of each community is determined by majority voting based on the ground truth species information of a predefined number of its trees. The species that receives the most votes is chosen as the final prediction for that community.

This approach enables tree species classification with minimal user input. In the best possible case, the user needs to classify only a single tree per community. If a fully automatic workflow is required, at least one template tree for each tree species has to be provided in advance. The ground truth species information is propagated to the remaining trees within the same communities. While using majority voting, the users may find communities containing a mix of species. In this case, community detection workflow could be repeated with adjusted parameters, e.g., an increased $\alpha$ or $\beta$ would generate more communities, which might prevent the undesirable merging of different species.

## Results and discussion

The proposed framework was verified for species clustering performance and classification accuracy. Specifically, the proposed features were analysed in terms of species separability, and the impact of relevant parameters $\alpha$ and $\beta$ to clustering accuracy was analysed. The proposed framework was compared with the established clustering methods. Next, an ablation study was conducted to investigate the importance of the proposed features for clustering performance. Additionally, we examined the robustness of clustering to varying point cloud density, voxel size $\delta$, and tree orientation. We also systematically addressed outlier detection within the datasets. Finally, we evaluated the proposed method’s classification accuracy by comparing it with two reference methods: an SVM trained on the proposed features and a PointConv neural network applied directly to point clouds.

### Data Sources

**Figure 4:**
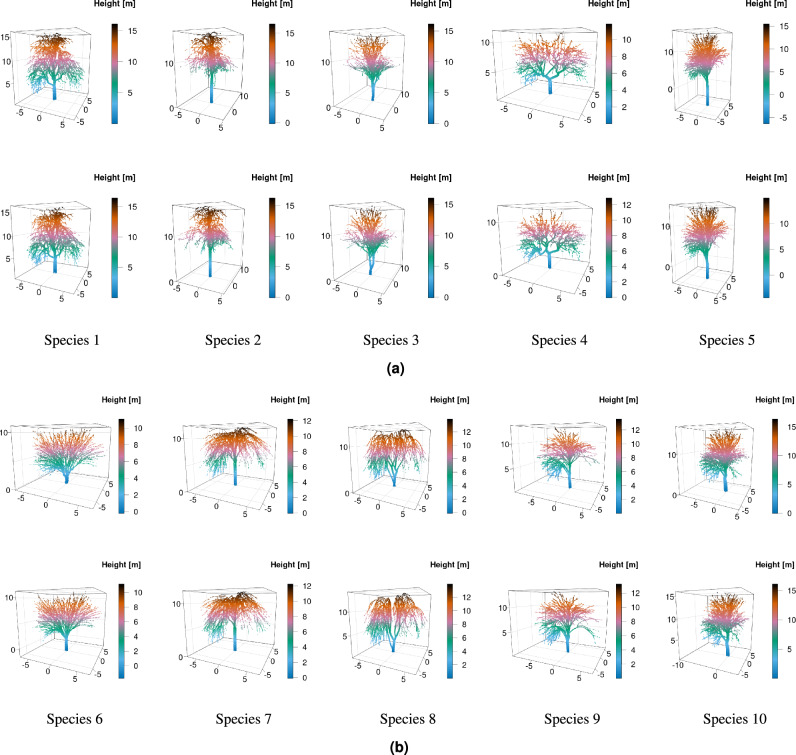
Examples of synthetic point clouds of (**a**) species 1 to 5 and (**b**) species 6 to 10, where each column represents a different species, and the second row shows additional trees of the same species.

To ensure the reproducibility of the proposed framework, verification was performed on two publicly available point clouds of individual trees with known species^51,56^. The first dataset was a synthetic one, containing artificially generated tree point clouds^51^. This dataset includes point clouds of 100 trees, synthesized algorithmically based on the predefined parameters of 10 species^57^, with 10 trees per species. These trees are visually diverse and consistent with their assigned species. The synthetic dataset minimises the potential for bias, e.g., due to measurement errors in real-world plots, and therefore makes analysis clearer. Fig. 4 show the distinct species present in the synthetic point cloud, demonstrating high tree similarity within the same species.

**Figure 5:**
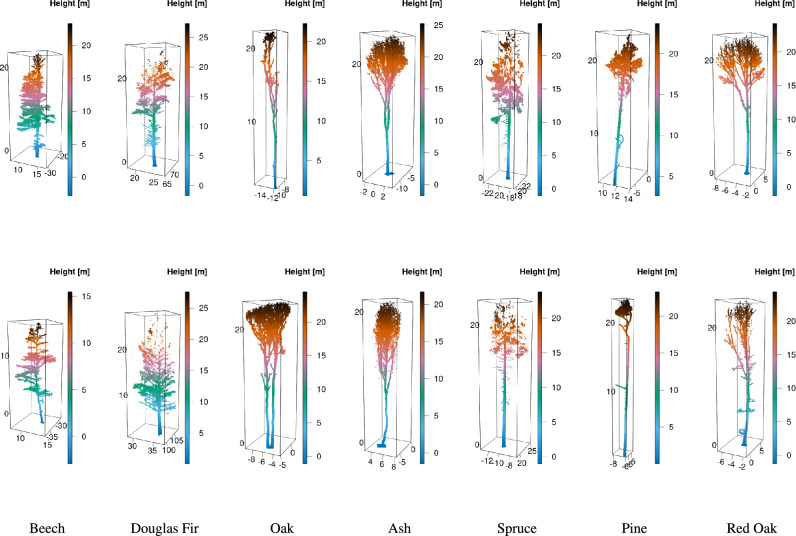
Examples of real-world point clouds^56^, where each column represents different species, and the second row shows additional trees of the same species.

Synthetic datasets, while useful, often don’t reflect the inherent noise and measurement errors that are present in the real-world data, which cause the algorithms to underperform in practical use. To verify the framework on real-world data, we utilised the publicly available dataset of point clouds generated from a terrestrial LiDAR scanning campaign^56^. This dataset consists of 691 trees across 7 species: 1. Beech (164 trees), 2. Douglas Fir (183 trees), 3. Oak (22 trees), 4. Ash (39 trees), 5. Spruce (158 trees), 6. Pine (25 trees), and 7. Red Oak (100 trees). The dataset has been used previously to validate tree species classification based on supervised machine-learning^42^, allowing for direct comparison with the proposed framework. The dataset is described in the same study^42^ in more detail. The point clouds were collected from a variety of managed and unmanaged forest sites in Germany and the United States (Oregon) using either a Faro Focus 3D 120 (Faro Technologies Inc., Lake Marry, FL, United States) or a Zoller and Fröhlich Imager 5006 (Zoller and Fröhlich GmbH, Wangen i.A., Germany) scanner^42^. Fig. 5 demonstrates the distinct species in the real point clouds, while also highlighting the visual diversity among the trees of the same species.

### Analysis of features

**Figure 6:**
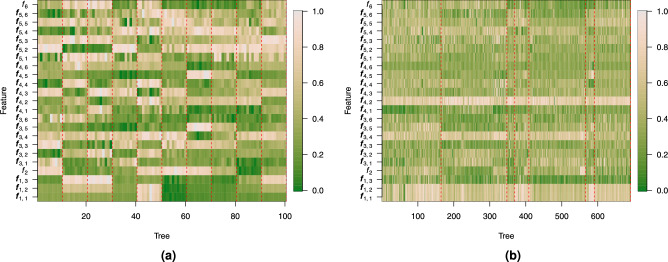
Normalized feature components of individual trees in (**a**) synthetic and (**b**) real-world datasets. The vertical dashed lines represent the boundaries between ground truth species of tree samples. Therefore, the tree samples within the vertical boundaries belong to the same species. Species are ordered the same as in Figs. 4 and 5.

Before validating tree species separability, we analysed the derived features. We examined a total of 23 feature components per tree, grouped into six feature sets ($\vec {f}_1$ to $f_6$) as follows: $\vec {f}_1$ (3 feature components), $f_2$ (1), $\vec {f}_3$ (6), $\vec {f}_4$ (6), $\vec {f}_5$ (6), ${f}_6$ (1). For visualisation purposes, each feature component was normalized independently into the range [0,1]. The normalized feature components for both synthetic and real-world datasets are shown in Fig. 6.

**Figure 7:**
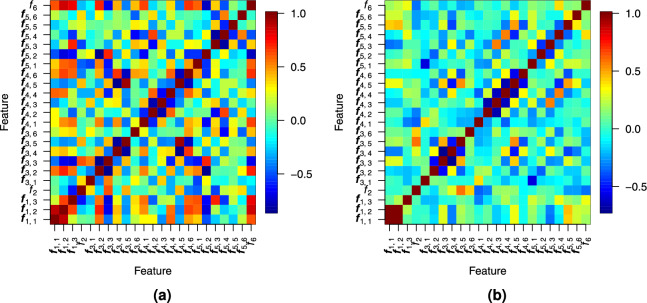
Pearson correlation between the feature components of trees in (**a**) synthetic and (**b**) real-world datasets.

A distinction of individual feature component values is evident across tree species in both cases. However, this distinction is clearer in the synthetic data because the variance of feature components in the real-world dataset is higher. While each feature can distinguish between some tree species, no single feature can distinguish between all of them. Fig. 7 shows that the correlation between most feature components is low.

This suggests that different features provide complementary information, which facilitates species separation.

### Species separability evaluation

To evaluate quantitatively the tree species separability/clustering performance of the proposed framework, we measured the similarity between the predicted and ground truth clusterings. We used the Rand Index (RI), a common metric ranging from 0 to 1. However, RI is known to be biased towards higher values, even for random results. Therefore, we also employed the Adjusted Rand Index (ARI)^58^, which ranges from -1 to 1, and is adjusted to give a score of 0 for random clustering and higher value for good species separability.

**Figure 8:**
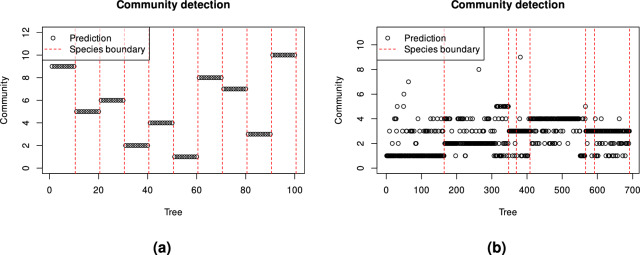
Species clustering performance of the proposed framework on (**a**) simulated point clouds and (**b**) real-world captured point clouds. The circles indicate the species clusters assigned to individual trees, while the vertical dashed lines represent the boundaries between ground truth species of trees. Therefore, the trees within the vertical boundaries belong to the same species. Species are ordered in the same way as in Figs. 4 and 5.

Fig. 8a shows the tree species separability for the simulated trees using our proposed approach.

The resulting RI and ARI are 1.00 and 1.00, respectively. These results demonstrate the practical usability of the proposed framework, as the predicted clusters aligned perfectly with the ground truth clusters.

Meaningful but less homogeneous clusters were achieved on the real-world dataset as shown in Fig. 8b. The achieved RI and ARI were 0.757 and 0.295, respectively. These results were achieved with the same parameters as for synthetic point clouds. The achieved ARI value denotes that the community detection performed better than random chance, while still showing a significant amount of disagreement. This represents an acceptable score with potential for improvements. Overall, the detected communities concur with real-world species while some trees were placed in the wrong communities and some species were merged into a common community. The most pronounced merging is that of species 3, 4, 6 and 7 (Oak, Ash, Pine, and Red Oak). A closer visual inspection of the point clouds reveals two challenges: some trees of different species look very similar (e.g., the bottom Oak tree and the upper Ash tree in Fig. 5), while some trees of the same species can be quite different structurally (e.g. Oak trees in Fig. 5). Therefore, achieving a perfect classification accuracy on these datasets is difficult even visually.

**Figure 9:**
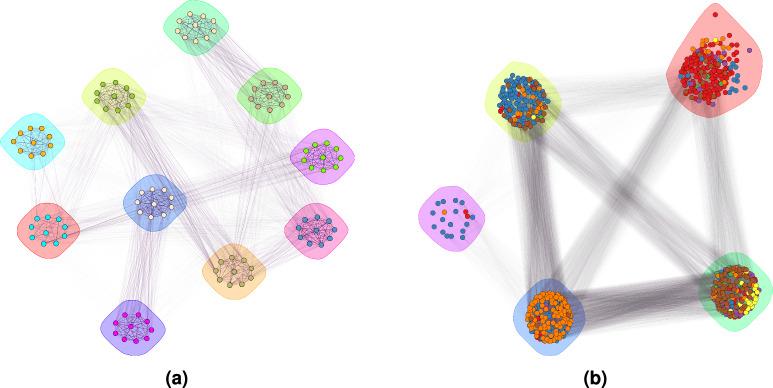
Community detection results of the proposed framework on (**a**) simulated point clouds, and (**b**) real-world captured point clouds.

Community detection within the proposed framework supports good interpretability of the results. This can be confirmed by Fig. 9.

Each node represents an individual tree, and the colour of the nodes indicates the ground truth species. The intensity of the edges is proportional to the weights (inverse distances) between the nodes.

A perfect match between the detected communities and actual species (node colours) relates to perfect performance of the proposed framework on simulated data. However, on real-world data, the approach had lower performance, although each community still featured a prevalent species. As already previously confirmed, closer visual examination revealed significant shape differences among same-species trees assigned to different communities, possibly due to varying growth conditions, while some trees of different species were grouped together due to high similarity. This discrepancy raises the application-dependent question of whether to fuse or split such communities.

**Figure 10:**
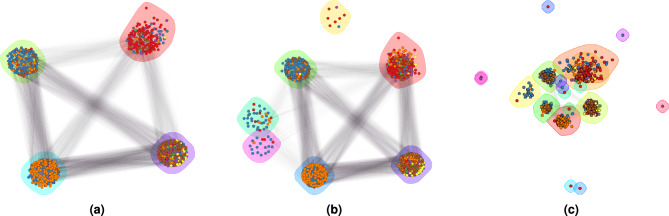
Community detection results of the proposed framework on real-world captured point clouds with (**a**) $\alpha$ = 1.6 (RI = 0.751, ARI = 0.293), (**b**) $\alpha$ = 2.4 (RI = 0.752, ARI = 0.248), and (**c**) $\beta$ = 0.9 (RI = 0.756 and ARI = 0.209).

Separability of species could be increased with different values of $\alpha$ and $\beta$ parameters. While the need to tune these parameters for different datasets might seem like a limitation, it gives the user direct control over the separability of species. For example, a more homogeneous forest would require higher values of $\alpha$ and $\beta$ parameter to distinguish similar trees, whereas a more heterogeneous forest would not. The proposed framework allows the user to increase or decrease this separability, while the default parameter values ($\alpha$ = 2 and $\beta$ = 0.1) are useful on both datasets. Fig. 10 shows community detection results for the following parameter settings: $\alpha$ = 1.6, $\alpha$ = 2.4, and $\beta$ = 0.9.

**Figure 11:**
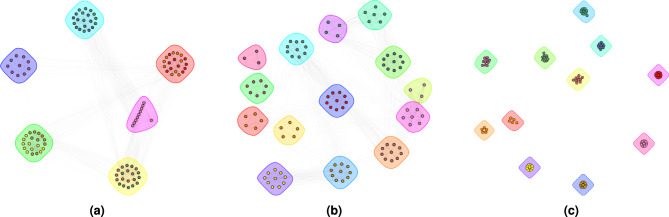
Community detection results of the proposed framework on synthetic point clouds with (**a**) $\alpha$ = 1 (RI = 0.982, ARI = 0.878), (**b**) $\alpha$ = 10 (RI = 0.919, ARI = 0.651), and (**c**) $\beta$ = 0.92 (RI =0.995, ARI = 0.969).

A similar impact of changed $\alpha$ and $\beta$ is evident in the synthetic dataset (Fig. 11).

The values of $\alpha$ and $\beta$ clearly modulate the number of communities, where datasets with more homogeneous trees (e.g., synthetic dataset) require larger changes in $\alpha$ and $\beta$ to change the number of communities.

**Figure 12:**
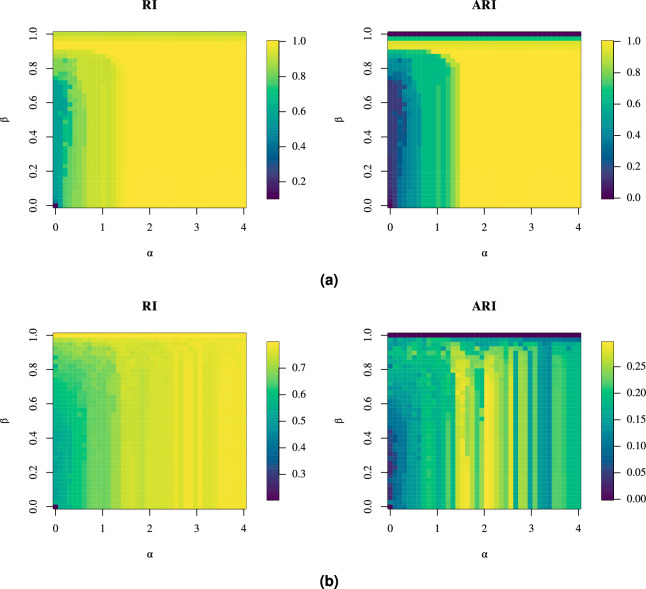
The impact of $\alpha$ and $\beta$ on community detection performance (RI and ARI values) of the proposed framework for (**a**) synthetic and (**b**) real-world point clouds.

Figure 12 illustrates a more detailed evaluation of the impact of $\alpha$ and $\beta$ on the results, where $\alpha$ was varied from 0 to 4, and $\beta$ from 0 to 1, with a step size adjusted to yield 40 steps for each parameter.

The most distinct results were observed for the synthetic dataset. Here, the highest accuracy was achieved with $\alpha$ around 2 or with higher values of $\beta$. Specifically, larger $\beta$ values removed more edges, which artificially created sub-graphs that aided community detection. When $\alpha = 0$, all edge weights were set to 1, meaning that only the graph topology determined that connectivity for community detection. In this case a higher $\beta$ value around 0.975 ensured high accuracy. Therefore, parameters $\alpha$ and $\beta$ offer flexibility, as some community detection algorithms do not support weighted edges.

For the real-world dataset, the influence of $\beta$ was smaller while the optimal value of $\alpha$ was again around 2. The highest $\beta$ values had detrimental effect on accuracy. Incorporating weights into community detection with $\alpha > 0$ improved the accuracy. Overall, $\alpha = 2$ and $\beta < 0.9$ are good compromise values for both datasets.

#### Comparison with the reference clustering methods

In terms of usability, the proposed community-based method has two important advantages over classical clustering methods: better interpretability and no requirement to specify the number of communities in advance. This enables community detection in heterogeneous forests with various outliers. To put the performance of the proposed framework into perspective, it was compared to the reference clustering methods included in the programming language R: K-means clustering with Hartigan-Wong distances^59^, DBScan clustering using a kd-tree^60^, and Ward’s hierarchical agglomerative clustering^61^. Clustering was performed directly on feature vectors $F^{(t)}$ of each tree *t*. In addition, the Leiden community detection algorithm^62^ was included in the comparison with the same default parameter values $\alpha = 2$ and $\beta = 0.1$.

The stochastic algorithms (K-means clustering and Leiden) were executed 101 times with median, min and max values of RI and ARI reported. For DBScan, Ward’s hierarchical clustering, and Leiden community detection, the best parameters according to ARI were found for each dataset using a grid search as follows. The resolution parameter of the Leiden algorithm was tuned across a range of 0.00025 to 0.4 with a step size of 0.00025. For DBScan, the neighbourhood radius was optimized from 1 to 20 with a step size of 0.2, while the minimum number of points required in the neighbourhood was tuned from 0 to 20 with a step size of 1. The cut height for Ward’s hierarchical clustering was set in the range of 1 to 40 with a step size of 0.1. All other algorithms used their default parameter settings.

The results in Table 1 demonstrate significantly lower performance for K-means clustering on the same features as the proposed framework, even with the predefined appropriate number of clusters.

**Table 1:** Performance of reference clustering algorithms on the features in relation to the proposed community detection-based framework.

	Simulated dataset		Real-world dataset	
Method	RI	ARI	RI	ARI
Hartigan-Wong (predefined number of clusters=10, median of 101 runs)	0.947	0.704	0.753	0.102
	[0.906–0.98]	[0.531-0.881]	[0.740–0.767]	[0.075–0.129]
Hartigan-Wong (predefined number of clusters=7, median of 101 runs)	0.915	0.629	0.729	0.100
	[0.759–0.933]	[0.423–0.68]	[0.692–0.748]	[0.052–0.143]
DBScan (radius of the $\epsilon$ neighbourhood=3, number of minimum points required in the $\epsilon$ neighbourhood=0)	0.939	0.631	0.796	0.004
DBScan (radius of the $\epsilon$ neighbourhood=5, number of minimum points required in the $\epsilon$ neighbourhood=12)	0.515	0.137	0.556	0.111
Ward’s hierarchical clustering (cut height=17)	0.985	0.912	0.796	0.038
Ward’s hierarchical clustering (cut height=38.1)	0.871	0.513	0.791	0.063
Leiden community detection (resolution=0.013, median of 101 runs)	0.995	0.969	0.792	0.150
	[0.975–0.995]	[0.858–0.969]	[0.782–0.795]	[0.094–0.172]
Leiden community detection (resolution=0.0085, median of 101 runs)	0.939	0.718	0.782	0.176
	[0.939–0.939]	[0.718–0.718]	[0.770–0.787]	[0.108–0.192]
Fast greedy modularity optimisation algorithm	**1**	**1**	**0.757**	**0.295**

**Figure 13:**
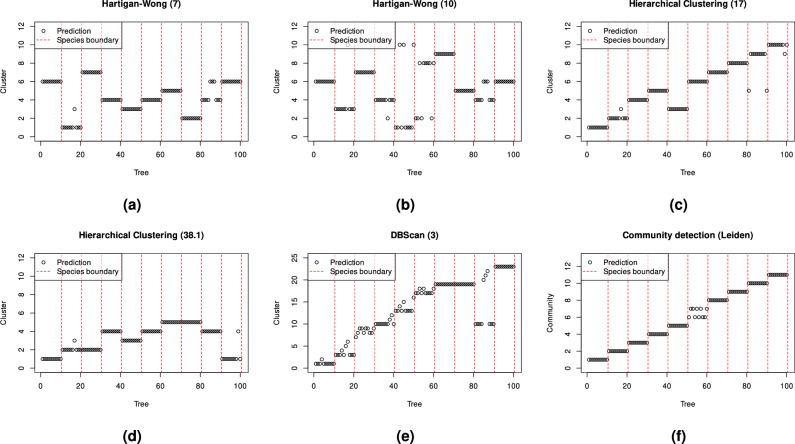
Examples of tree species separability on synthetic point clouds of (**a**) K-means clustering with 7 clusters (**b**) the K-means clustering with 10 clusters (**c**) Hierarchical clustering with cut height=17 (**d**) Hierarchical clustering with cut height=38.1, (**e**) DBScan with radius of the $\epsilon$ neighbourhood=3 and number of minimum points required in the $\epsilon$ neighbourhood=0, and (**f**) Leiden’s community detection with a resolution equal to 0.013. The vertical dashed lines represent boundaries between the ground truth clusters, while the circles represent species clusters for individual trees.

**Figure 14:**
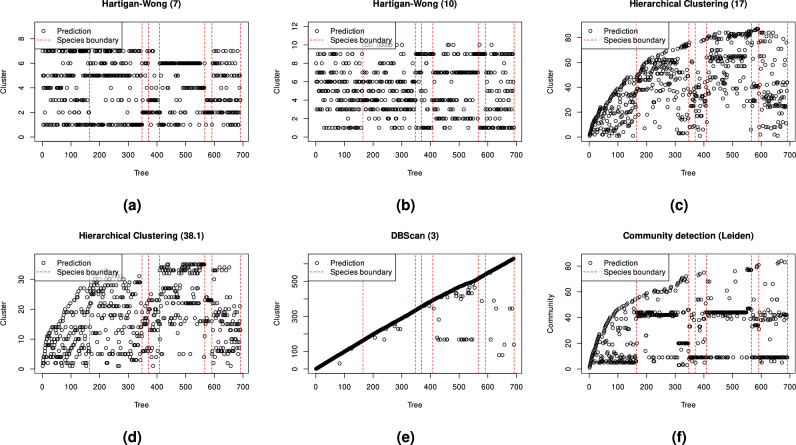
Examples of tree species separability on real-world point clouds of (**a**) K-means clustering with 7 clusters (**b**) the K-means clustering with 10 clusters (**c**) Hierarchical clustering with cut height=17 (**d**) Hierarchical clustering with cut height=38.1, (**e**) DBScan with radius of the $\epsilon$ neighbourhood=3 and number of minimum points required in the $\epsilon$ neighbourhood=0, and (f) Leiden’s community detection with a resolution equal to 0.0085. The vertical dashed lines represent boundaries between the ground truth clusters, while the circles represent species clusters for individual trees.

The one-sided t-test showed that reference clustering methods had significantly lower accuracy with p-values consistently below $10^{-10}$. The only exception was the RI accuracy of Leiden algorithm which achieved a higher accuracy on real-world dataset with a p-value again below $10^{-10}$. Furthermore, a typical drawback of RI is evident, as values become inflated with a higher number of clusters (e.g., DBScan with a radius of the $\epsilon$ neighbourhood=3). These quantitative results are consistent with the visual examples of species separability on synthetic point clouds in Fig. 13 and real-world point clouds in Fig. 14.

Given that the number of clusters was predetermined to match the number of species, potentially favouring community detection, these results show the highest possible potential of classical clustering methods. However, the set parameter values are not sufficient for achieving good clustering performance, as shown in Figs. 13 and 14. DBScan, on the other hand, works without a predefined number of clusters^63^, but requires other parameters that impact performance significantly. The best parameters are not the same for different datasets. Similarly, hierarchical clustering works without a predefined number of clusters, but still requires cutting the tree (i.e., a dendrogram) at a given height, with a high impact on the tree species clustering performance. Leiden community detection within the proposed framework with appropriate parameters shows competitive results compared to the used fast greedy modularity optimisation algorithm^55^ on both datasets.

### Ablation study

To verify the impact of individual features on tree species clustering performance, we performed an ablation study by running the proposed framework on a reduced set of features (i.e., limited exhaustive feature elimination). Tables 2 and  3 shows clustering performance with one or two features removed, for both simulated and real-world datasets.

**Table 2:** (a) RI and (b) ARI values of the proposed approach on the synthetic dataset when removing individual features from the full set ($\vec {f}_1$ to $f_6$).

	$\vec {f}_1$	${f}_2$	$\vec {f}_3$	$\vec {f}_4$	$\vec {f}_5$	${f}_6$
(a)						
$\vec {f}_1$	1					
${f}_2$	0.936	0.955				
$\vec {f}_3$	0.96	0.934	0.98			
$\vec {f}_4$	0.96	0.913	0.899	0.98		
$\vec {f}_5$	0.996	0.923	0.943	0.953	0.981	
${f}_6$	1	0.953	0.965	0.963	0.966	0.984
(b)						
$\vec {f}_1$	1					
${f}_2$	0.7	0.766				
$\vec {f}_3$	0.796	0.681	0.889			
$\vec {f}_4$	0.796	0.615	0.588	0.889		
$\vec {f}_5$	0.977	0.647	0.715	0.754	0.888	
${f}_6$	1	0.756	0.81	0.802	0.815	0.903

Diagonal values represent the removal of a single feature, while off-diagonal values correspond to the removal of a pair of features from the full set.

**Table 3:** (a) RI and (b) ARI values of the proposed approach on the real-world dataset when removing individual features from the full set ($\vec {f}_1$ to $f_6$).

	$\vec {f}_1$	${f}_2$	$\vec {f}_3$	$\vec {f}_4$	$\vec {f}_5$	${f}_6$
(a)						
$\vec {f}_1$	0.745					
${f}_2$	0.639	0.699				
$\vec {f}_3$	0.685	0.675	0.700			
$\vec {f}_4$	0.679	0.673	0.681	0.755		
$\vec {f}_5$	0.693	0.678	0.658	0.669	0.714	
${f}_6$	0.652	0.667	0.647	0.666	0.655	0.699
(b)						
$\vec {f}_1$	0.253					
${f}_2$	0.145	0.186				
$\vec {f}_3$	0.145	0.157	0.157			
$\vec {f}_4$	0.170	0.174	0.172	0.289		
$\vec {f}_5$	0.240	0.148	0.120	0.174	0.195	
${f}_6$	0.134	0.149	0.112	0.169	0.124	0.157

Diagonal values represent the removal of a single feature, while off-diagonal values correspond to the removal of a pair of features from the full set.

The results indicate that all features contribute to species clustering performance, but $f_2$, $\vec {f}_3$, and $f_6$ appear to be the most important features. Surprisingly, the PCA-derived feature $\vec {f}_1$ was not among the most important ones.

As the next experiment, we have executed the proposed framework on a reduced set of features by using only one or two features at a time (i.e., limited exhaustive feature selection). The results in Tables 4 and 5 indicate that using one or two features is not sufficient for high separability accuracy.

**Table 4:** (a) RI and (b) ARI values of the proposed approach on the synthetic dataset when using only one or two features from the full set ($\vec {f}_1$ to $f_6$).

	$\vec {f}_1$	${f}_2$	$\vec {f}_3$	$\vec {f}_4$	$\vec {f}_5$	${f}_6$
(a)						
$\vec {f}_1$	0.589					
${f}_2$	0.714	0.576				
$\vec {f}_3$	0.661	0.814	0.579			
$\vec {f}_4$	0.838	0.717	0.79	0.592		
$\vec {f}_5$	0.755	0.737	0.828	0.818	0.529	
${f}_6$	0.622	0.742	0.654	0.771	0.583	0.577
(b)						
$\vec {f}_1$	0.135					
${f}_2$	0.263	0.172				
$\vec {f}_3$	0.205	0.409	0.151			
$\vec {f}_4$	0.409	0.287	0.372	0.178		
$\vec {f}_5$	0.284	0.309	0.425	0.397	0.134	
${f}_6$	0.165	0.296	0.201	0.332	0.16	0.147

Diagonal values represent the results of using a single feature, while off-diagonal values show the results of using a pair of features.

**Table 5:** (a) RI and (b) ARI values of the proposed approach on the real-world dataset when using only one or two features from the full set ($\vec {f}_1$ to $f_6$).

	$\vec {f}_1$	${f}_2$	$\vec {f}_3$	$\vec {f}_4$	$\vec {f}_5$	${f}_6$
(a)						
$\vec {f}_1$	0.511					
${f}_2$	0.575	0.538				
$\vec {f}_3$	0.595	0.606	0.558			
$\vec {f}_4$	0.54	0.543	0.579	0.522		
$\vec {f}_5$	0.533	0.55	0.572	0.552	0.547	
${f}_6$	0.585	0.587	0.556	0.565	0.542	0.559
(b)						
$\vec {f}_1$	0.027					
${f}_2$	0.05	0.076				
$\vec {f}_3$	0.1	0.109	0.103			
$\vec {f}_4$	0.031	0.079	0.134	0.013		
$\vec {f}_5$	0.076	0.073	0.123	0.101	0.088	
${f}_6$	0.135	0.098	0.118	0.126	0.074	0.118

Diagonal values represent the results of using a single feature, while off-diagonal values show the results of using a pair of features.

The strongest individual features in synthetic dataset are $f_2$ and $\vec {f}_4$. In the real world dataset, the two most important features are $\vec {f}_3$ and $f_6$.

### Robustness to rotation and resolution

**Figure 15:**
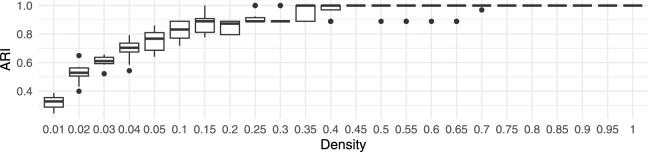
Boxplot of the ARI for different densities of point cloud in the synthetic dataset.

To verify the robustness of the proposed framework, clustering performance was verified across different point cloud densities. Original point clouds were decimated from 100% to 1% of the initial points using uniform random sampling. At each density level, the experiment was repeated ten times with newly decimated point clouds. The results in Fig. 15 demonstrate the robustness of the proposed framework, as the clustering performance variance within individual densities remained relatively small.

Therefore, random sampling had minimal impact on the performance.

**Figure 16:**
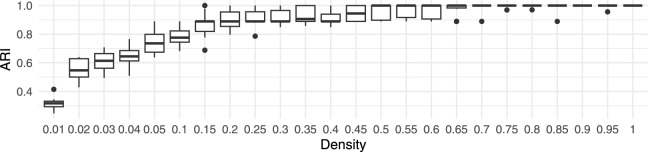
Boxplot of the ARI for different densities of point cloud in the synthetic dataset, where random rotations between 0 and $2 \pi$ radians around the central vertical axis were applied to individual point clouds.

Another important aspect of the proposed framework is its robustness to the orientation of point clouds. To verify this, the input point clouds were rotated randomly around the central vertical axis, with a uniform distribution between 0 and $2 \pi$ radians. The results in Fig. 16 show species clustering performance when the individual point clouds were rotated randomly.

The experiment was performed 10 times (each time with a different random rotation), and Fig. 16 in comparison to Fig. 15 demonstrates low ARI variance within the individual densities, indicating the high robustness of the proposed framework to point cloud orientation. Furthermore, a comparison with Fig. 15 shows high similarity, indicating that orientation did not increase variability in clustering performance significantly. This is especially important when performing tree species clustering on larger diverse datasets from multiple regions, where environmental impacts due to shading can produce asymmetric tree crowns.

**Figure 17:**
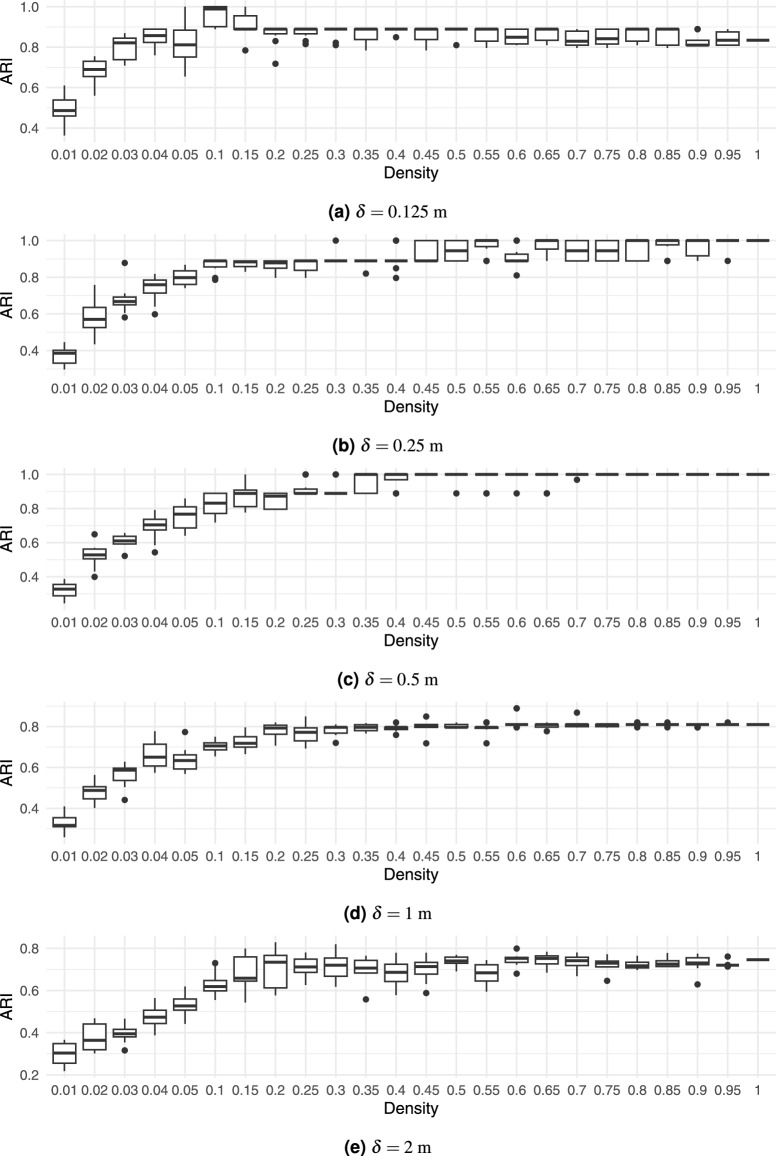
Boxplots of the ARI for different point cloud densities in the synthetic dataset at various voxel sizes $\delta$.

Finally, the impact of voxel size on robustness was tested by performing feature extraction at different densities and voxel sizes $\delta$ of 0.125 m, 0.25 m, 0.5 m (default), 1 m, and 2 m, while repeating the process 10 times. The framework’s robustness is shown by comparing in Fig. 17a the ARI at the default voxel size ($\delta$=0.5 m) to the ARI at different voxel sizes. Large voxel sizes of 2 m obscured the details of the tree shape, and thus degraded the performance significantly.

Very small voxels also decreased the performance, because they increased the grid sparseness, amplifying sensitivity to smaller variations in the point clouds. Additionally, smaller voxels increased the processing and memory resources.

### Detection of outliers

An important application of the proposed methodology is a clear and reliable identification of outliers for manual review. We tested the outlier detection by adding individual trees one by one from one dataset (i.e., potential outliers) to another dataset. After we calculated the weights between the trees using Eq. (7), we performed outlier detection in the feature space as follows. For each tree, we calculated the mean weight of connections to other trees. Next, for each tree, we determined the rank of its mean weight in the sorted list of all trees’ mean weights. A low rank indicated that a potential outlier tree has a smaller mean weight and was therefore unrelated to other trees.

**Figure 18:**
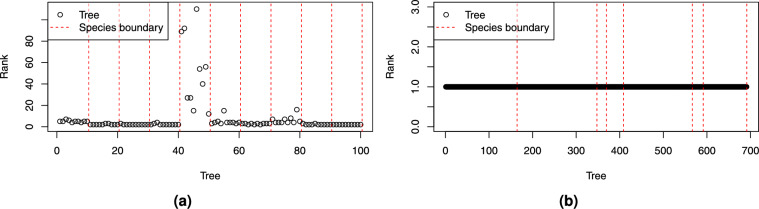
Rank of mean weights for outlier trees (circles) from (**a**) synthetic trees injected into the real-world dataset, and from (**b**) real-world trees injected into the synthetic dataset.

Fig. 18a illustrates the rank of mean weights for outlier trees injected from the synthetic into the real-world dataset.

The results for synthetic outliers show clear patterns, indicating that most species can be classified as outliers already in the feature space without community detection. The only exceptions are species 5 and, partly, species 8. In the case of real-world outlier, there is a clear pattern: all trees had rank 1. This means that every tree from the real-world dataset was very different in the feature space from trees in the synthetic dataset. This is expected, as trees in synthetic dataset are more homogeneous and similar to each other, while the real-world trees are more distinct and such outliers are trivial to identify.

**Figure 19:**
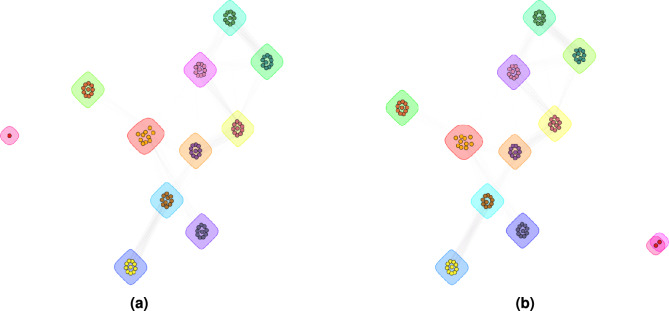
Visual community detection results on synthetic dataset with (**a**) one injected real-world tree as outlier (sole circle) and (**b**) two injected real-world trees as outliers (two sole circles).

Community detection can provide a more holistic approach to outlier detection by placing outliers into smaller communities. To achieve this, we executed the community detection algorithm on top of the graphs from the previous paragraph. Fig. 19 clearly shows outlier trees from the real-world dataset (Beech species) within communities of a synthetic dataset.

**Figure 20:**
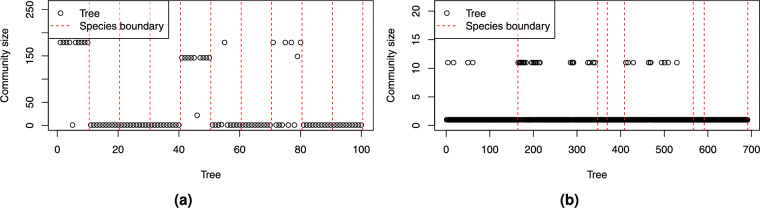
Size of the community at $\beta$ = 0.7 containing the injected outlier tree (circle): for trees (**a**) from synthetic dataset injected into the real-world dataset, and for trees (**b**) from real-world dataset injected into the synthetic dataset.

To isolate the outliers, it was sufficient to set $\beta$ to 0.7, which reduced the tendency of outlier nodes to form edges with other nodes and be merged into larger communities. The results of all the trees from both datasets are shown in Fig. 20.

For each injected outlier tree from the source dataset, we counted how many trees from the destination dataset belonged to the same community. The results of injecting synthetic trees into the real-world dataset (Fig. 20) are consistent with Fig. 18, with the exception of species 1, which tends to infiltrate large communities. In the reverse case, where real-world trees were injected into the synthetic dataset, a small proportion of trees were joined with existing synthetic tree communities of size 10 (Fig. 20b).

### Classification accuracy

The main aim of the proposed framework is efficient tree species classification that does not require extensive tuning of machine learning models or manual preparation of training datasets. To classify tree species using the proposed method, we randomly selected a predefined number of trees from each community and labelled the selected trees based on ground truth data. We then performed majority voting to determine the species of other trees in each community.

We report the classification results only for the real-world dataset^42^ because the synthetic dataset is straightforward to classify due to perfect community detection. That is, in the synthetic dataset, only one tree from each of the 10 communities needs to be labelled manually to classify all trees automatically with $100\%$ accuracy.

**Figure 21:**
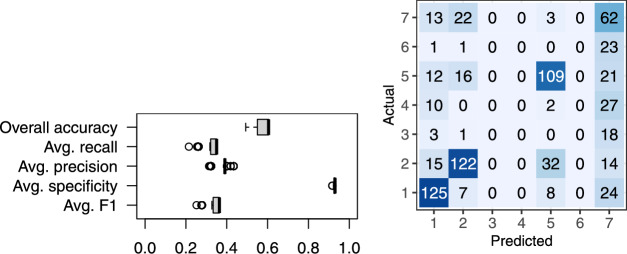
(**a**) Box plot of tree species classification accuracy over 101 trials with 15 manually labelled trees per community and (**b**) confusion matrix from the median trial, based on overall accuracy.

Fig. 21 shows the confusion matrix for real tree species classification with 15 manually labelled trees per community, achieving an overall accuracy of 60.5%.

The overall accuracy was calculated as the proportion of all correctly classified trees. Because overall accuracy can be misleading with imbalanced classes, we include other metrics for evaluation of the model’s performance. Therefore, we also report the macro-average recall, which is the average of per class recall values for each class. Similarly, we calculate the average precision over each class, average specificity, and the average F1-score, which represents the harmonic mean of precision and recall. These metrics are particularly useful because they give equal weight to each class, preventing a model’s performance from being dominated by the majority class.

**Figure 22:**
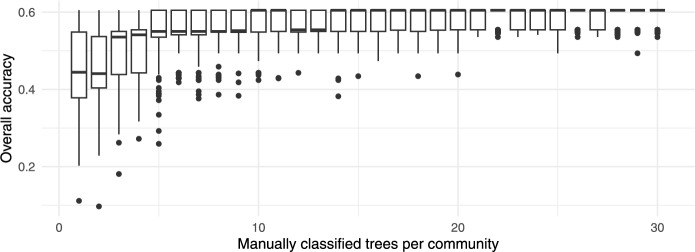
Box plot of the overall classification accuracy in relation to the number of manually labelled trees per community.

The performance of the proposed semi-automated classification approach depends on the species distribution and the number of trees sampled from each community. Fig. 22 shows the overall classification accuracy with respect to the number of manually labelled trees in each community, where the sampling was repeated 100 times.

As expected, the overall classification accuracy improved with the increasing number of manually labelled trees per community, converging to a final value of 60.5%. A higher number of trees also decreased the variance of the overall classification accuracy. While a higher number of labelled trees per community improved the overall classification accuracy, this improvement still depends on the community detection performance. With good species separability, only a small number of trees within each community need to be classified. The proposed approach detected 5 communities, which is the main cause for lower accuracy. These communities were assigned species labels via majority vote. The assigned labels corresponded to only 4 of the 7 ground-truth species, indicating that some species were never a majority in any community.

The proposed approach is suitable as a semi-automatic tree species classification tool, and is competitive, even to supervised machine learning methods. Seidel et al.^42^ reported 86.01% and 80.2% overall accuracy using a convolutional neural network on rendered images of the same trees with and without data augmentation, respectively. Although, in this case, the machine learning-based approach achieved higher overall accuracy, it used training and test datasets from the common regions. In contrast, the proposed framework does not require extensive training dataset preparation, making it more suitable in cases where learning datasets are not available, especially in new regions, as it requires minimal user input. By processing the point clouds directly with PointNet, Seidel et al.^42^ achieved about 48% overall accuracy, which is lower than our proposed approach.

#### Classification accuracy of SVM on the proposed features

To directly validate the suitability of the proposed features for tree species classification, we employed a supervised machine learning SVM method to classify tree species based on these features. We used a radial basis kernel and the default parameters of the e1071 package for R programming language^1^. In the first step, we split our dataset into training and testing subsets using a uniform sampling method to ensure that each species was represented proportionally. We performed experiments using three different train:test split ratios: 10:90, 20:80, and 80:20. For each split ratio, we trained the SVM model on the training data and then evaluated its classification accuracy on the testing data. This process was repeated 101 times. The different split ratios were used to assess the model’s ability to generalize with varying amounts of training data.

**Figure 23:**
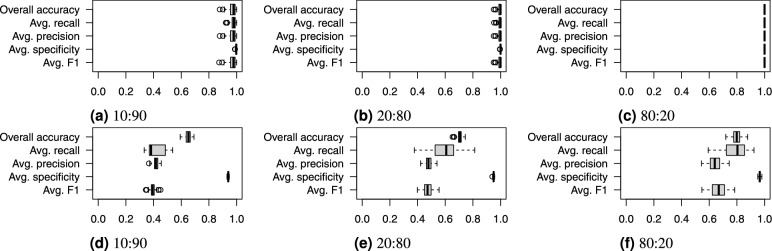
Boxplots of SVM classification accuracy on (**a**, **b**, **c**) the synthetic and (**d**, **e**, **f**) the real-world datasets using train:test dataset splits of 10:90, 20:80, and 80:20.

**Figure 24:**
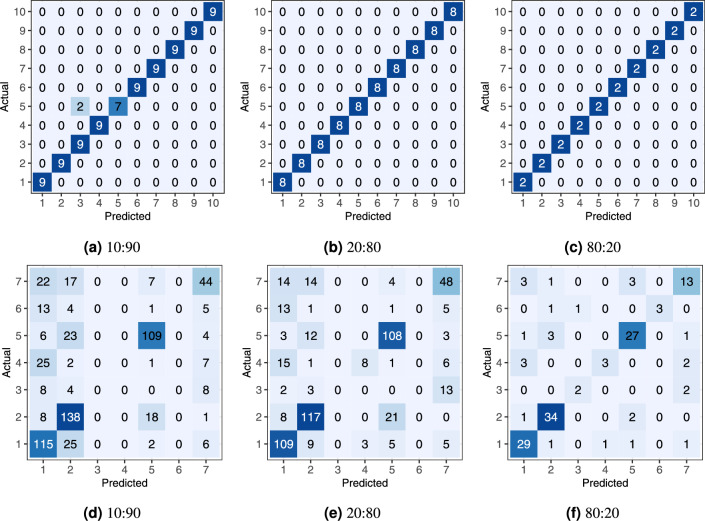
Confusion matrices of median SVM classification accuracy on (**a**, **b**, **c**) the synthetic and (**d**, **e**, **f**) the real-world datasets using train:test dataset splits of 10:90, 20:80, 80:10.

Fig. 23 shows the boxplots of the classification accuracy for the synthetic and real-world datasets with all three split ratios, while Fig. 24 presents the confusion matrix for the SVM models with the median overall accuracy.

On the synthetic dataset, the features allowed for nearly perfect classification, as shown in Fig. 23. The 10:90 split, which represents a limited training dataset, lowered the overall accuracy to approximately 98%.

On the real-world dataset, the SVM demonstrated significantly lower accuracy. The 80:20 split, which is commonly used in similar studies, resulted in an overall accuracy of approximately 80%. This is comparable to the 86.01% and 80.2% accuracies reported by Seidel et al.^42^, who used a convolutional neural network on rendered tree images. We can conclude that, the derived feature vectors are useful for tree species classification as they describes 3D tree shape sufficiently for tree species classification task. Smaller training datasets with 20:80 and 10:90 splits caused a notable drop in overall accuracy, falling into the range between 60% and 70%.

The confusion matrix in Fig. 24 for the real-world dataset reveals specific misclassification patterns. The most problematic species to classify were Oak (species 3), Ash (species 4), and Pine (species 6). Misclassifications were not random; the problematic species were frequently confused with Beech (Species 1) and Red Oak (Species 7) across all split ratios.

#### Comparison with PointConv

We compared the proposed framework with PointConv^20^ implementation^2^ which we adapted for the used datasets. The modified implementation is appended in the supplementary material. The code, originally designed for the ResNet dataset, accepts spatial coordinates with additional attributes (e.g., normals or colour information), resulting in six values per point (x, y, z, and colour/normal). Since our datasets lack additional attributes and point coordinates are the most widely available attributes in LiDAR data, we removed additional inputs for this comparison while preserving the original architecture. Although this modification prevents demonstrating the full potential of the PointConv, it ensures a baseline for the comparison.

The training configuration was as follows: the maximum number of training epochs was 400 and 500 for synthetic and real-world datasets, respectively. The selected optimizer was AdamW^64^ with batch size of 16. The learning rate was experimentally determined to be 0.001. The number of sampled points per input was 1024. During training we shuffled points and performed transformations of point clouds (i.e., random rotations of trees around the vertical axis, translations of trees by 20%, scaling of trees by 25%, random dropout of points with a random ratio from 0% to 80%) as a data augmentation technique to mitigate model overfitting.

**Figure 25:**
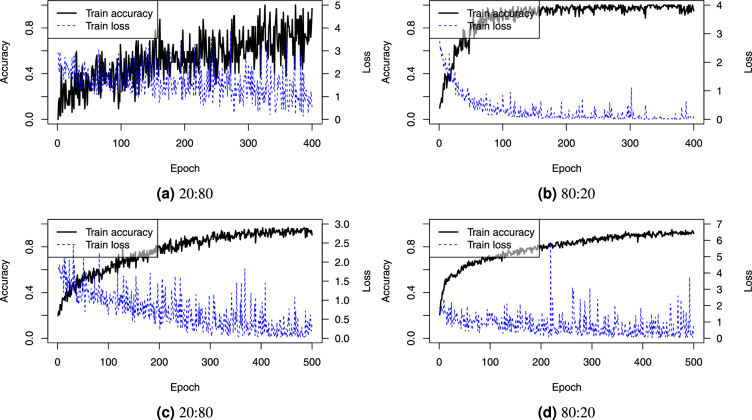
PointConv training overall accuracy over epochs of the best-performing models on (**a**, **b**) synthetic and (**c**, **d**) real-world on train datasets, with train:test splits of (**a**, **c**) 20:80 and (**b**, **d**) 80:20.

We assessed classification accuracy by splitting the dataset into training and testing subsets using stratified sampling to ensure that each species was represented in similar proportions in both subsets. The experiments were performed using two different split ratios (20:80 and 80:20) to verify the performance impact of smaller training datasets. For each split ratio, we repeated the training and testing process 11 times. The convergence curves in Fig. 25 demonstrate that training was run for a sufficient number of epochs.

**Figure 26:**
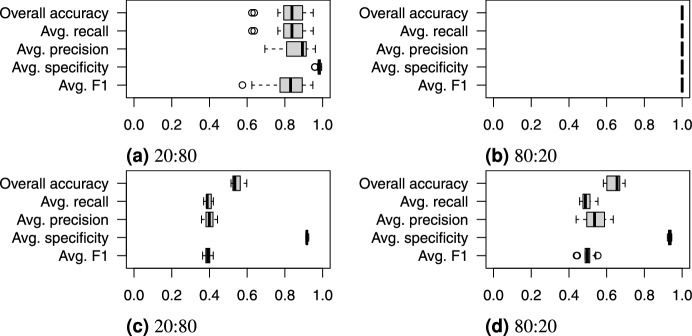
Boxplot of PointConv performance on (**a**, **b**) the synthetic and (**c**, **d**) the real-world datasets on train:test split of (**a**, **c**) 20:80 and (**b**, **d**) 80:20.

**Figure 27:**
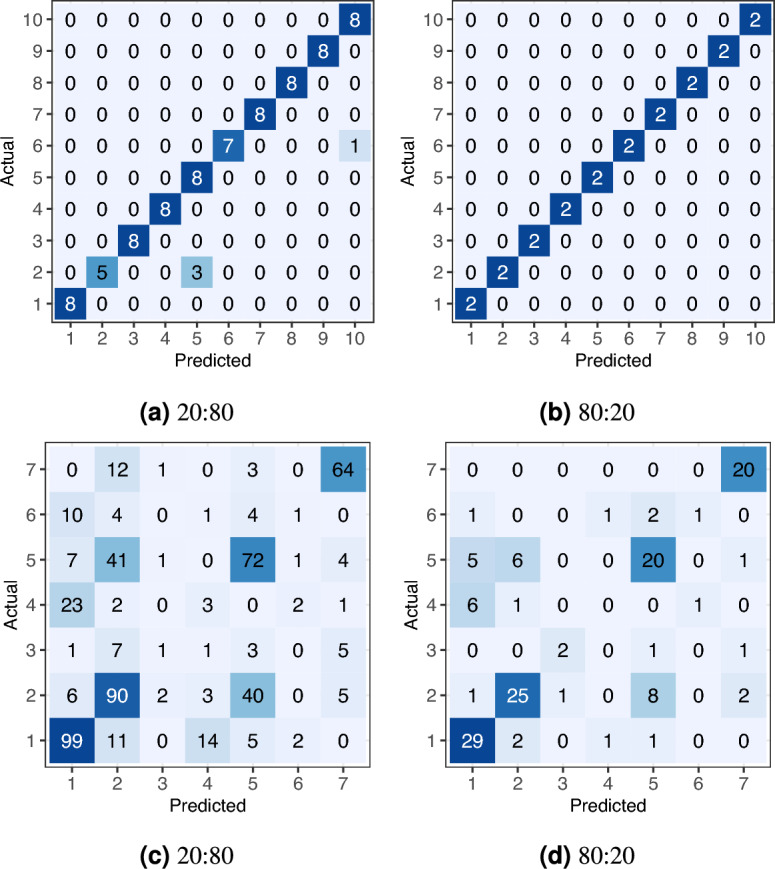
Confusion matrices for the best trained PointConv models on (**a**, **b**) the synthetic and (**c**, **d**) the real-world test sets for train:test splits of (**a**, **c**) 20:80 and (**b**, **d**) 80:20.

Fig. 26 shows a box plot of the achieved performance on the test sets, while Fig. 27 shows the confusion matrix of the best trained model evaluated on the training set.

On the synthetic dataset, overall accuracy and average recall values are the same due to the equal number of trees in each class. PointConv achieves perfect accuracy with an 80:20 split, but the performance degrades with a smaller training dataset (i.e., a 20:80 split). This is expected, since complex models with many parameters require larger training datasets in order to avoid overfitting while data augmentation can only partially mitigate this issue.

Overall accuracy on the real-world dataset was significantly higher than the 48% achieved by the PointNet model from Seidel et al.^42^. This finding concurs with other studies^20,43^ demonstrating an improvement in accuracy in the range of 20%. However, this accuracy is still lower than the 80% achieved by the SVM working directly on the proposed features and the CNN on tree images by Seidel et al.^42^. Although a possible solution is tuning the model architecture for the specific datasets, we have preserved the original architecture and instead used only data augmentation to mitigate overfitting. The most challenging species to classify were the same as with the SVM, i.e., Oak (species 3), Ash (species 4), and Pine (species 6). These findings again concur with the PointNet results by Seidel et al.^42^. Finally, the average recall was notably lower due to the unequal representation of species.

The results highlight a common limitation of traditional machine learning models, which require larger training datasets. PointConv was previously tested on significantly larger datasets (e.g., ShapeNet and ModelNet40 have 16,881 and 12,311 models, respectively)^20^ while our datasets consist of only hundreds of trees. For diverse forests, creating comprehensive training data can be impractical. Therefore, data augmentation techniques are one of the possible mitigation tools while architecture fine-tuning has the potential to improve the results even further. Finally, the proposed methodology offers a good alternative while the SVM trained with the proposed features on smaller datasets is capable of achieving competitive performance.

## Conclusion

This paper proposes a new framework for unsupervised tree species clustering and classification, which is conceptually different than the widespread supervised machine learning techniques in this domain. The proposed approach combines feature extraction on individual trees with graph-based community detection. The core innovations lie in the development of new voxel-based features on point clouds that exhibit robustness to rotation and variations in point cloud density. We fused multiple features further, which were in different scales, into one unified measure of tree dissimilarity. Subsequent community detection on the constructed graph grouped trees by species effectively, yielding good species separability, especially on synthetic data. Leveraging these community assignments, we also propose a semi-automatic tree species classification method requiring minimal user intervention.

The proposed approach is competitive with state-of-the-art machine-learning-based tree species classification methods, as demonstrated by the comparison with PointConv. We have also demonstrated that the proposed features provide good separability of species, which allowed the SVM to achieve competitive classification performance to PointConv. These findings suggest that the proposed features are a strong foundation for future work on other datasets. To ensure broad applicability, this validation was focused on point coordinates since it represents the most consistent and widely available attribute in LiDAR data.

A key advantage of our community-based classification is its inherent interpretability, facilitated by clear graphical representations that enable the visual identification of outliers and anomalies, such as an unexpected number of communities. This visual feedback allows for early detection of potential performance issues. Moreover, our framework is not intended to replace machine learning, but rather to enhance it by providing efficient means of generating initial training datasets with minimal manual annotation, thus serving as a valuable data preparation tool. Although the method’s accuracy on the real-world dataset is not high enough for immediate use of the results, it offers a foundation by partially pre-labeling a significant portion of the training data or even the whole dataset. This makes it easier for users to check and correct the output rather than perform a full manual classification from scratch.

The generality of our framework, allowing for the seamless integration of new features, ensures its immediate applicability for other researchers. By employing publicly accessible datasets and sharing the processing code used, this paper establishes a good foundation for future advancements in tree species clustering and classification. Future research could explore the validation of our approach on more comprehensive datasets, incorporating ortho-photo imagery, full waveform LiDAR data, and additional point attributes (e.g., colours), where automated feature extraction could expedite the progress significantly. As PointConv supports additional point attributes it remains a perfect baseline for comparison with additional types of data and to demonstrate the full potential of the PointConv.

Additional future research directions include the development of more efficient algorithms for deriving complex features, with automatic feature extraction representing a promising path forward. Additionally, the accuracy of community-based classification could be improved further through iterative community splitting based on the manual identification of mixed-species communities and the incorporation of new discriminating parameters. The modular design of our framework supports efficient evaluation of new features by focusing specifically on the clustering performance.

## Supplementary Information

Supplementary Information.

## Data Availability

Programming code and data generated or analysed during this study are included in this published article (and its Supplementary Information files). The supplementary material is additionally deposited in the repository (DOI: 10.6084/m9.figshare.29136395). The supplementary material contains: - Derived feature vectors of individual trees in the JSON file format. - Code in the programming languages C++ and R to derive the features. - Code in the programming language R to process features to detect communities of trees. - Modified PointConv model for tree species classification on publicly available datasets.

## Footnotes

1 https://CRAN.R-project.org/package=e1071

2 https://github.com/DylanWusee/pointconv_pytorch
